# Successful Hemostasis With Platelet Transfusion and Tranexamic Acid, and Inhibitor Elimination With Cyclosporine, in Steroid-Resistant Acquired Coagulation Factor V Deficiency Caused by Antibiotic Readministration

**DOI:** 10.1155/crh/1402790

**Published:** 2025-07-08

**Authors:** Kazuto Togitani, Moe Yamamoto, Soichiro Tanaka, Rei Aono, Yoshiki Uemura

**Affiliations:** ^1^Department of Hematology, Chikamori Hospital, Kochi, Japan; ^2^Department of Gastroenterology, Chikamori Hospital, Kochi, Japan; ^3^Department of Cardiology, Chikamori Hospital, Kochi, Japan

**Keywords:** acquired factor V inhibitor, antibiotics, cefepime, cyclosporine, platelet transfusion, tranexamic acid

## Abstract

Acquired factor V deficiency (AFVD) is a rare coagulation abnormality associated with infectious diseases, antibiotics, surgery, autoimmune diseases, and malignancy, which causality is difficult to prove. Here, we report a case of a 90-year-old woman who developed melena following antibiotic treatment for pneumonia. She had been on cefepime for bacterial pneumonia for 2 months to 2 weeks prior to her arrival in the emergency room. Upon presentation, she had severe anemia (Hb: 6.7 g/dL) and prolonged PT (74.3 s) and activated partial thromboplastin time (APTT) (161.9 s). Coagulation studies revealed incomplete correction of the APTT in a 1:1 mixing study with normal pooled plasma, factor V activity of 0%, and a factor V inhibitor titer of 13 Bethesda units, confirming the diagnosis of AFVD. Since the antibiotics were not recognized as the cause, the coagulation abnormality worsened after their readministration. The melena subsequently improved with platelet transfusion and administration of tranexamic acid, while prednisolone-resistant coagulation abnormalities improved with cyclosporine A (CsA) treatment. This case shows the importance of avoiding suspected drugs and the effectiveness of CsA as a second-line treatment of AFVD.

## 1. Introduction

Acquired factor V deficiency (AFVD) is a rare coagulation abnormality. Its incidence was reported as 0.038 cases per million people per year in a Japanese nationwide survey [1], similar to that in Singapore (0.09) [2] and less than that in Australia (0.23) [3]. It is noticed when both prothrombin time (PT) and activated partial thromboplastin time (APTT) are elevated in the absence of disseminated intravascular coagulation (DIC), chronic liver disease or excessive anticoagulant use, etc. [1]. It is caused by autoantibodies to coagulation factor V (FV), which almost always belongs to the IgG class and less often to the IgA class, [1]. The symptoms of AFVD vary widely from being asymptomatic to fatal hemorrhage or, rarely, thrombosis [1, 2, 4–6]. Historically, the correlation between bovine thrombin and the causality of AFVD has been well defined [7, 8] although it is no longer used in clinical practice. Other than bovine thrombin, infectious diseases, antibiotics, surgery, autoimmune diseases, hemodialysis, and malignancy have been described as associated underlying diseases or conditions related to AFVD [1, 2, 4–6]. Idiopathic cases have also been reported in 21%∼28.9% of the patients [1, 2, 4–6]. In the clinical setting, the causality of AFVD cases is difficult to prove because surgery, infection, and antibiotic therapy frequently occur sequentially and/or simultaneously in patients with AFVD.

We experienced an AFVD case in whom the disease developed after antibiotic administration and worsened after its readministration, seeming like an antigenic boost. Although prednisolone (PSL) did not improve prolongation of PT and APTT in this patient, cyclosporine A (CsA) administration led to improvement of the coagulation abnormality. This case report suggests the importance of realization and avoidance of the possible iatrogenic causes of AFVD, especially antibiotics, and demonstrates the efficacy of CsA in PSL-resistant AFVD cases, in addition to the hemostatic efficacy of platelet transfusion and tranexamic acid.

## 2. Case Description

**Figure 1:**
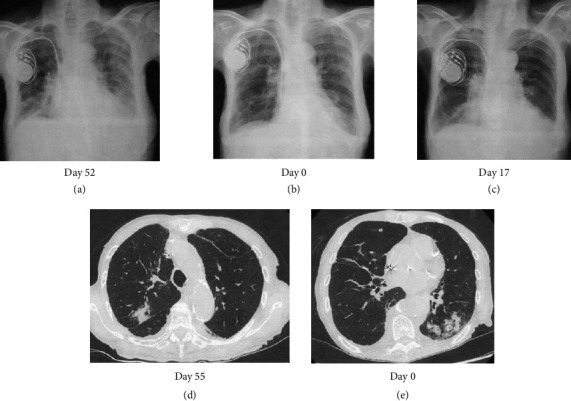
Chest X-ray before (a) and after (b) onset and before resurgence (c) of AFVD showed questionable lung infiltration. Chest CT before (d) and after (e) onset of AFVD clearly showed lung infiltration compatible with bacterial pneumonia. The number of days is calculated starting from the time of the patient's current hospitalization.

**Figure 2:**
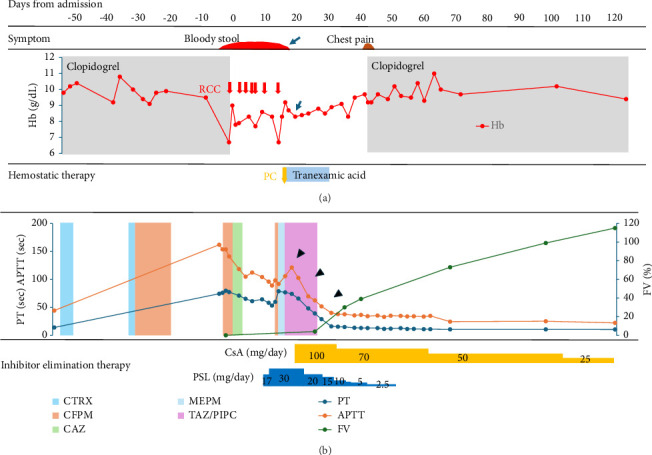
Clinical course. The (a) shows hemostasis therapy, symptoms and Hb levels, and the (b) shows changes in inhibitor removal therapy, PT, and APTT. The bar chart shows the timing of clopidogrel and antibiotic administration. PC; platelet concentrate, RCC; red cell concentrate, CsA; cyclosporine A, PSL; prednisolone, CTRX; ceftriaxone, CFPM; cefepime, CAZ; ceftazidime, MEPM; meropenem, TAZ/PIPC; tazobactam/piperacillin, PT; prothrombin time, APTT; activated partial thromboplastin, FV; factor V.

A 90-year-old female visited the emergency room with difficulty in moving, left lower abdominal pain, and bloody stools for 3 days and was admitted with a tentative diagnosis of gastrointestinal bleeding, bacterial pneumonia (Figures 1(b) and 1(e)), and anemia. Her medical history was positive for complete atrioventricular block that was treated with pacemaker insertion, angina pectoris, and chronic thyroiditis although she had no history of bleeding. She had received ceftriaxone (CTRX) and cefepime (CFPM) intermittently from 2 months to 2 weeks earlier for bacterial pneumonia (Figures 1(a) and 1(d)) due to *Serratia marcescens*, which is a β lactamase-producing bacterium. Her clinical course is shown in Figure 2. Her current medications included clopidogrel, esomeprazole, azosemide, spironolactone, rosuvastatin, benfotiamine, pyridoxine hydrochloride, cyanocobalamin, dapagliflozin, trichlormethiazide, sacubitril valsartan, and levothyroxine. None of these oral medications have been changed recently.

Physical evaluation indicated a performance status of 4, blood pressure of 71/46 mmHg (130/63 mmHg after fluid replenishment), pulse rate of 80 beats/minutes, body temperature of 36.5°C, SpO_2_ of 99% (under nasal oxygen supplementation), tenderness in the left lower abdomen without muscular defense, and bloody stools on rectal examination.

Her laboratory data on admission indicated severe anemia with an Hb of 6.7 g/dL and prolongation of PT to 74.3 (reference range: 10.0–13.0) seconds and APTT to 161.9 (reference range: 20.0–40.0) seconds (Table 1). Fibrinogen, fibrin degradation products (FDP), D-dimer level, and platelet count were within the normal range. Since there was no evidence of active bleeding on CT imaging, emergency endoscopy was not performed. We supplemented vitamin K to correct the coagulation abnormalities although it did not improve the prolongation of PT and APTT. Lupus anticoagulant testing by the diluted Russell viper venom time method was inconclusive since there was no coagulation both before and after neutralization of phospholipids. Anticardiolipin antibodies and anti-β2 glycoprotein I antibodies were negative. A 1:1 mixing study of the patient's plasma and normal pooled plasma did not correct the APTT immediately (APTT: 150.7 s) or following 120 min of incubation (APTT: 159.2 s), indicating the presence of a circulating fast-acting inhibitor unlike acquired factor VIII inhibitor. In addition, her FV activity was reduced to 0%, and the level of FV inhibitor (FVI) was 13 Bethesda Units/mL, indicating autoantibodies to FV. Although the levels of coagulation factors II, VII, IX, and X were low, an increase in the activity of these factors after dilution was observed, indicating a falsely low level of these coagulation factors caused by FVI in one stage coagulation assay. Thus, a diagnosis of AFVD was made. Since investigations for autoantibodies and CT imaging excluded autoimmune diseases, except Hashimoto's thyroiditis, and neoplasia as triggering factors for the FVI, we considered pneumonia (Figures 1(b) and 1(e)) as a possible trigger for FV inhibition at this point.

Since the patient's general condition stabilized after red blood cell transfusion, and prolongation of PT and APTT gradually improved, clopidogrel was discontinued, and she was observed without any other treatment in the hope of natural recovery, with reference to recommendations from the literature [9].

Thereafter, CFPM, which was successful at the time of the previous hospitalization for bacterial pneumonia, was administered again (Day 0∼Day 3), although it was changed to ceftazidime (CAZ), since the *Stenotrophomonas maltophilia* identified in sputum was found to be sensitive to this drug, and it was discontinued on Day 6 due to normalization of the inflammatory response. However, since anemia and melena requiring red blood cell transfusion continued after discontinuing clopidogrel, we decided to administer PSL 0.5 mg/kg (=17 mg) from Day 13, along with platelet transfusion and tranexamic acid (3 g/day). With this therapy, the melena gradually subsided and anemia did not worsen after Day 18 (arrow in Figure 2). However, since her PT and APTT prolongation did not improve with this therapy, the dose of PSL increased to 30 mg from Day 16. Since fever was observed from Day 17, CFPM administration was again started empirically based on a tentative diagnosis of pneumonia (Figure 1(c)). However, the patient developed hypoxemia and hypotension on Day 18, considering a CFPM-induced drug hypersensitivity reaction, CFPM was discontinued, and meropenem (MEPM) and subsequently tazobactam piperacillin (TAZ/PIPC) were administered due to the development of MEPM-induced prurigo, with gradual improvement in her pneumonia.

Despite increasing the dose of PSL, prolongation of PT and APTT continued to worsen again after Day 16 in our patient, indicating steroid-resistant AFVD. Since the use of CsA for acquired coagulation factor deficiency is an off-label treatment in Japan, we obtained informed consent from the patient and her family to administer CsA. Thereafter, 100 mg CsA (equivalent to 3 mg/kg) was commenced on Day 23, resulting in marked improvement in the patient's symptoms. Simultaneously, her PT and APTT decreased (arrowheads in Figure 2), and FVIs disappeared on Day 45.

Meanwhile, on Day 41, the patient developed chest pain. Although changes in the ECG could not be determined due to pacemaker insertion, a mild increase in troponin T levels was observed. Although recurrence of angina was suspected, percutaneous coronary angioplasty was not performed due to her age and poor performance status. After consulting with a cardiologist, clopidogrel was resumed, resulting in the disappearance of her chest pain.

Since PT and APTT prolongations improved after the start of CsA, PSL was tapered and terminated, and she was transferred to a rehabilitation hospital on Day 60. CsA was gradually reduced, with no further flare-up of FVI to date.

## 3. Discussion

AFVD is suspected in patients with prolongation of PT and APTT with no history of bleeding disorder in the patient or family, in whom disseminated DIC, and chronic liver disease are excluded, and when the condition is not corrected by mixing the patient's plasma with normal plasma [1]. Although the hepaplastin test (HPT) and thrombotest (TT) are useful for estimating FV deficiency, it could not be performed in this case because of its unavailability due to lack of insurance approval in Japan [9]. Therefore, in this case, the diagnosis could not be made until the results of FV analysis were obtained. Since the patient's general condition was stabilized by blood transfusion despite gastrointestinal bleeding, and prolongation of PT and APTT seemed to be reducing spontaneously (Figure 2), steroid treatment was postponed, and her progress was monitored with only discontinuation of clopidogrel in the hope of spontaneous remission [9].

Even after the diagnosis of AFVD is confirmed by the decrease in FV activity and a positive FVI result, we did not realize that the cause of FVI was antibiotics and instead attributed it to pneumonia (Figures 1(b) and 1(e)). We only retrospectively noticed that CFPM was the culprit of FVI based on the facts that PT and APTT prolongation had occurred about 2 weeks after the first CFPM administration and had recurred as PSL resistant following the third CFPM administration with CFPM-induced hypersensitivity reaction.

A literature search of previous reviews of AFVD, excluding those due to bovine thrombin exposure, dated 1955–2010 [5], 2010–2016 [6], and 2016–2020 [10], demonstrated that antibiotics were reported to be associated with the AFVD in 42% (33/78) [5], 32% (15/47) [6], and 34% (5/12) of the cases [10], respectively. Subsequently, we performed a PubMed search of case reports in the previous 5 years using the search terms “acquired factor V inhibitors” and “acquired coagulation inhibitors.” Initially, 27 new cases were found from among 19 published case reports during 2020–2024 [11–29], including two Japanese case series [9, 23]. To ensure continuity with previous studies, two cases involving bovine serum were excluded [9], resulting in 25 new cases on PubMed from 2020 to 2024 that were analyzed (Table 2). Among them, the most common association was with antibiotics (60%, 15 out of 25) (Table 2).

Recently, two nationwide surveys were published from France [4] and Japan [1]. Among 38 AFVD cases diagnosed in France between 1988 and 2015, exposure to antibiotics was the most frequent cause of AFVD (57.9%; 22 out of 38 cases) [4]. On the other hand, among 24 patients diagnosed in Japan between 2016 and 2020, along with 177 AFVD cases published from Japan, 19.4% (39 out of 201) of the patients with AFVD had infectious diseases (mainly involving the respiratory system) and 16.9% (34 out of 201) of the patients were administered antibiotics [1]. Therefore, in the old case reports in Japan, events such as antimicrobial use, which were judged to be less related to the occurrence of AFVD, might have been underreported. Pathophysiologically, antibodies produced against bacterial cell walls in relation to antibiotics might aberrantly bind to the C2 domain of FV [10] and inflammation, such as secondary to major surgery or pneumonia, might trigger an autoimmune reaction to coagulation factors, including FV, as has been demonstrated by proteome analysis [29]. Although not few (15%; 5/33 cases [5]) antibiotic-related FVI reportedly disappeared spontaneously [5, 30], there have been a few reports of deaths, including in antibiotic readministration cases [5, 31]. These data outline the importance of careful observation of the clinical course of AFVD, identification of suspected drugs, and avoidance of secondary exposure as soon as possible [4, 5, 31].

No trials exist comparing the treatment options for AFVD due to its rare presentation. Basically, the management of AFVD involves a two-step approach, consisting of controlling bleeding and eradicating the inhibitor [8]. Bleeding in patients with FVI might be difficult to treat since FV concentrates are not available and FVIs interfere with the prothrombinase complex and are difficult to bypass using prothrombin complex concentrate (PCC) or recombinant factor VIIa [1, 8]. Cases of effective hemostasis following platelet transfusion have been increasingly reported [4, 5]. Physiologically, since 20% of FV is stored in platelets and FV in platelets can escape inhibitors, platelet transfusion is thought to be effective for hemostasis in AFVD [9]. On the other hand, the success rate of platelet transfusion in Japan (26.7% (8/30)) was lower than in previous global reports (68.8% (11/16)) [1, 5, 6]. Although differences in dosage and duration of administration of platelets have been noted, the reasons for the lower efficacy of platelet transfusion on AFVD in Japan are not well understood. In our patient, 10 units of platelet transfusion resulted in an improvement of melena. We also administered the antifibrinolytic, tranexamic acid, with reference to British guidelines [32]. Although tranexamic acid was reportedly used in only 1% of patients with AFVD in Japan because of concerns about the nonhyperfibrinolytic state [1], the concomitant transfusion of platelets together with tranexamic acid in this case might have had synergistic effects since the transfused platelets would have provided an adequate amount of FV needed to ensure successful fibrin plug formation, and tranexamic acid would have inhibited dissolution of the fibrin plugs by inhibiting plasmin at the sites of gastrointestinal bleeding. Hence, we postulate that tranexamic acid might be useful in the hemostatic procedure. However, it should be noted that tranexamic acid is relatively contraindicated in cases of urinary tract bleeding and in patients with a risk of blood clots [32]. Moreover, its use is absolutely contraindicated in fibrinolytic states associated with consumption coagulopathy, including conditions such as sepsis-induced coagulopathy [33].

Steroids are generally used as the first-line therapy for eliminating FVIs because of their high success rate [1, 4–6]. Other agents used include rituximab, intravenous immunoglobulin, plasmapheresis, cyclophosphamide, and CsA, with variable effects [6]. Among them, rituximab and cyclophosphamide should only be used in resistant cases because of the higher incidence of hematologic cytopenia and infection rates with their use [5, 6]. Since fewer side effects, such as cytopenia and infection, have been reported with CsA than with cyclophosphamide and rituximab, along with its quick response in cases of acquired hemophilia A, especially in elderly patients [34, 35], we used CsA for this patient with PSL-resistant AFVD. Initiation of CsA led to marked improvement of the patient's symptoms, with normalization of PT and APTT within 22 days. Although it cannot be ruled out that the FVIs would have spontaneously disappeared after CFPM discontinuation in this case, the rapid improvement in PT and APTT suggests that CsA was effective in eliminating FVI.

This case report has several limitations that should be acknowledged. First, this is a single case report, which limits the generalizability of our findings regarding the efficacy of CsA and the combination of platelet transfusion with tranexamic acid for AFVD management. Second, we could not definitively establish the causal relationship between CFPM and AFVD development, as the diagnosis was made retrospectively based on temporal associations rather than through rechallenge testing or other confirmatory methods. Third, the unavailability of HPT and TT in Japan due to insurance restrictions delayed our diagnostic process and may have affected treatment decisions. Fourth, we cannot completely exclude the possibility of spontaneous remission of FVI following CFPM discontinuation, making it difficult to definitively attribute the clinical improvement solely to CsA treatment. Fifth, the optimal dosing and duration of platelet transfusion and tranexamic acid combination therapy could not be established from this single case. Finally, long-term follow-up data regarding recurrence risk and potential complications of the treatment regimen are limited. These limitations highlight the need for larger prospective studies and standardized diagnostic approaches to better understand AFVD management strategies.

In conclusion, this report underscores the importance of identifying potential inciting drugs in the management of AFVD. In addition, this case demonstrates that CsA might be a useful treatment option, as an alternate to cyclophosphamide and rituximab, for inhibitor elimination in PSL-resistant cases. Furthermore, platelet transfusion and tranexamic acid might be useful options for hemostasis in AFVD.

## Figures and Tables

**Table 1:** Laboratory data on admission.

**Blood cell counts**	

WBC	7900/μL
RBC	186 × 10^4^/μL
Hemoglobin	6.7 g/dL
Hematocrit	20.5%
MCV	110.2 fL
Platelet count	21.1 × 10^4^/μL

**Serological tests**	

ANA	40
Anti-ds-DNAIgG Ab	< 10 IU/mL
Anti-SS-A Ab	< 1 U/mL
Anti-SS-B Ab	< 1 U/mL
Anti-CL-β2GP1 Ab	≤ 1.2 U/mL
Anti-CL Ab	< 4.0 U/mL
LA (dRVVT)	Not detectable

**Biochemistry tests**	

TP	5.9 g/dL
Alb	3.2 g/dL
T-bil	0.6 mg/dL
AST	28 U/L
ALT	27 U/L
LDH	243 U/L
ALP	72 U/L
γGTP	17 U/L
Cr	0.8 mg/dL
BUN	40.3 mg/dL
Na	138 mEq/L
K	4.1 mEq/L
Cl	101 mEq/L
CRP	0.44 mEq/L
Glu	115 mEq/L

**Coagulation tests**	
	**Reference range**

PT	74.3 (10∼13) s
PT-INR	6.55
APTT	161.9 (20∼40) s
Fibrinogen	336.6 (180∼350) mg/dL
FDP	≤ 2.5 (< 5.0) μg/mL
D-dimer	0.8 (≤ 1.0) μg/mL
FII: C	7 (75∼135)%
FV: C	0 (70∼135)%
FVII: C	59 (75∼140)%
FVIII: C	67 (60∼150)%
FIX: C	51 (70∼130)%
FX: C	39 (70∼130)%
FV-inhibitor	13 (undetected) BU/mL

**Table 2:** Literature review of acquired factor V deficiency from 2020 to 2024.

Study	Age (years)/gender	Hemorrhagic symptoms	Reported association	Inhibitor elimination therapy	Hemostatic therapy	Outcome
Ghachem et al. [11]	75/M	Arteriovenous shunt thrombosis	Cefotaxime, hemodialysis	Antibiotics discontinued		Remission

Vetri et al. [12]	68/F	Hematuria, hematomas (lower limbs, gluteus)	Idiopathic	PSL: 1 mg/kg and CPA: 100 mg/day	FEIBA	Remission

Matsumoto et al. [13]	68/M	Bleeding from hemodialysis sites	Hemodialysis, surgery, clindamycin, linezolid, rifampicin	PSL: 0.5 mg/kg	None	Remission

Yamada and Asakura [9]	74/M	Bloody stools	Rectal cancer	PEX	FFP, PC	No remission
	76/F	None	Cirrhosis, hepatocellular carcinoma, esophageal varices, radiofrequency ablation, ceftriaxone, levofloxacin	Antibiotics discontinued	None	Remission
	78/F	None	Cirrhosis, hepatocellular carcinoma, radiofrequency ablation, ceftriaxone	Antibiotics discontinued	None	Remission

Tochino et al. [14]	76/M	Intramuscular hemorrhage	Apixaban, levofloxacin	PSL: 20 mg/day	FFP	Remission

Bennett et al. [15]	87/F	Psoas muscle hematoma, retroperitoneal hematoma	COVID-19, levofloxacin, piperacillin/tazobactam	IVIG, PSL: 1 mg/g PEX	PC	Remission

Bruna et al. [16]	90/M	Hematuria	Surgery, amoxicillin-clavulanic acid	Steroid, CPA: 100 mg/day, RTX: 375 mg/m^2^, MMF: 2 g/day, VCR: 2 mg	FFP, rFVIIa, PCC	No remission

Hirata et al. [17]	43/M	None	Surgery, cefmetazole, meropenem	None	None	Remission

Pineau-Vincent et al. [18]	43/M	Gingival bleeding, bruises	Pernicious anemia	PSL: 1 mg/kg	None	Remission

Giuffrida et al. [19]	68/F	Hematoma, hematuria	Dabigatran	Dabigatran discontinued, mPSL: 1 mg/kg, CPA: 2 mg/kg	FFP, APCC	Remission

Yokota et al. [20]	71/F	None	Pancreatic cancer, sulbactam/cefoperazone, meropenem, levofloxacin	PSL: 0.5 mg/kg	None	Remission

Kida et al. [21]	64/M	None	Hypopharyngeal cancer, nivolumab, cefazolin	None	None	No remission

Sei et al. [22]	66/M	Nasal hemorrhage	IgG4-related disease	PSL: 50 mg	None	Remission
	71/M	Subdural hemorrhage, cerebellar hemorrhage	End-stage renal failure, PE, ceftriaxone	mPSL: 40 mg	FFP	No remission
	87/M	Subcutaneous hemorrhage around the eye	End-stage renal failure, DM	mPSL: 40 mg	FFP, PC, tranexamic acid	Remission
	78/M	None	S/o lymphoma	PSL: 30 mg	None	Remission
	87/M	Intracranial hemorrhage	Takotsubo cardiomyopathy, pneumonia, end-stage renal failure	mPSL: 40 mg	FFP	No remission

Katsuren et al. [23]	89/M	Bleeding from arteriovenous fistula	End-stage renal disease, congestive heart failure, pneumonia, ceftriaxone, ceftazidime, tolvaptan	PSL: 50 mg, ceftazidime and tolvaptan discontinued	None	Remission

Arakaki et al. [24]	70/M	Systemic bleeding	Hepatocellular carcinoma, surgery, atezolizumab, bevacizumab, cefmetazole, tazobactam/piperacillin, meropenem, daptomycin	PEX, RTX, PSL: 1 mg/kg	None	No remission

Yu et al. [25]	67/F	Venous thrombosis	Intracerebral bleeding, pneumonia, ceftriaxone	PEX, PSL: 50 mg	None	Remission

Ceglédi et al. [26]	35/F	Postoperative bleeding, gastrointestinal bleeding	Surgery	DEX, RTX, CPA, PEX, IVIG, CsA, sirolimus, bortezomib, MMF	rFVIIa, aPCC, FEIBA, PC, tranexamic acid	Remission

Ou et al. [27]	78/M	Gastrointestinal bleeding	Bullous pemphigoid	PSL	FFP	Remission

Ansari et al. [28]	50/M	Bleeding from injection site and tracheostomy	COPD, HT, DM, schizoaffective disorder, COVID-19, tracheostomy, gastrostomy, *Enterococcus faecalis* urinary tract infection, carbapenem-resistant *Pseudomonas aeruginosa* isolated from tracheal aspirate, ceftriaxone, azithromycin, ampicillin, tobramycin	IVIG, mPSL: 40 mg, RTX	FFP, PC, tranexamic acid	Remission

*Note:* PSL: prednisolone, CPA: cyclophosphamide, PEX: plasma exchange, IVIG: intravenous immunoglobulin, RTX: rituximab, MMF: mycophenolate mofetil, VCR: vincristine, DEX: dexamethasone, CsA: cyclosporine A, HT: Hypertension, COVID-19: Coronavirus disease 2019, mPSL: methylprednisolone.

Abbreviations: APCC, activated prothrombin complex concentrate; COPD, chronic obstructive pulmonary disease; DM, diabetes mellitus; FEIBA, factor eight inhibitor bypassing activity; FFP, fresh frozen plasma; PC, platelet concentrate; PCC, prothrombin complex concentrate; PE, pulmonary embolism; rFVIIa, recombinant factor VIIa.

## Data Availability

The datasets used and/or analyzed during the current study are not publicly available due to patient privacy and confidentiality but are available from the corresponding author upon reasonable request.

## References

[1] Ichinose A., Osaki T., Souri M. (2024). Diagnosis and Treatment of Autoimmune Acquired Coagulation Factor Deficiencies: An Evidence-Based Review of Japanese Practice. *Seminars in Thrombosis and Hemostasis*.

[2] Kuperan P., Ng C. H., Ng H. J., Ang A. L. (2009). Acquired Factor V Inhibitor. A Problem-Based Systematic Review. *Thrombosis and Haemostasis*.

[3] Favaloro E. J., Posen J., Ramakrishna R. (2004). Factor V Inhibitors: Rare or Not So Uncommon? A Multi-Laboratory Investigation. *Blood Coagulation and Fibrinolysis*.

[4] Goulenok T., Vasco C., Faille D. (2021). Acquired Factor V Inhibitor: A Nation-Wide Study of 38 Patients. *British Journal of Haematology*.

[5] Franchini M., Lippi G. (2011). Acquired Factor V Inhibitors: A Systematic Review. *Journal of Thrombosis and Thrombolysis*.

[6] Boland F., Shreenivas A. V. (2017). Acquired Factor V Inhibitors: A Review of Literature. *Annals of Hematology & Oncology*.

[7] Streiff M. B., Ness P. M. (2002). Acquired FV Inhibitors: A Needless Iatrogenic Complication of Bovine Thrombin Exposure. *Transfusion*.

[8] Knöbl P., Lechner K. (1998). Acquired Factor V Inhibitors. *Baillière’s Clinical Haematology*.

[9] Yamada S., Asakura H. (2020). [Acquired Factor V Inhibitor] (in Japanese). *Rinsho Ketsueki*.

[10] Chartier A. R., Hillert C. J., Gill H., Jha P. (2020). Acquired Factor V Inhibitor After Antibiotic Therapy: A Clinical Case Report and Review of the Literature. *Cureus*.

[11] Ghachem I., El Borgi W., Fekih Salem S. (2020). Haemodialysis Tunisian Patient With Acquired Factor V Inhibitor Associated to Arteriovenous Shunt Thrombosis. *Annales de Biologie Clinique*.

[12] Vetri D., Lumera G., Tarascio S. (2020). A Case of Acquired Factor V Deficiency in Patient With Bleeding. *TH Open*.

[13] Matsumoto A., Ogawa Y., Osaki T. (2020). [Successful Management of Acquired Factor V Deficiency Developing Shortly After Induction of Hemodialysis]. *Rinsho Ketsueki*.

[14] Tochino Y., Mushino T., Hori Y. (2020). [Acquired Factor V Inhibitor Associated With Apixaban]. *Rinsho Ketsueki*.

[15] Bennett J., Cunningham M. T., Howard C., Hoffmann M., Plapp F. V. (2021). Acquired Factor V Inhibitor in the Setting of Coronavirus Disease 2019 Infection. *Blood Coagulation and Fibrinolysis*.

[16] Bruna R., Moia R., Valpreda A. (2020). An Acquired Factor V Inhibitor Induced Uncontrolled Bleeding in a Postsurgery Patient. *Clinical Case Reports*.

[17] Hirata H., Sakurai Y., Takeda T., Kasetani T., Morita T. (2021). Development of Acquired Factor V Inhibitor After Surgical Procedure Without the Use of Fibrin Tissue Adhesives: A Case Report. *Cureus*.

[18] Pineau-Vincent F., Toulon P., Lemaire P., Laribi K. (2021). Acquired Factor V Inhibitor: Success of Steroids in a Patient With Primary Sclerosing Cholangitis, Ulcero Haemorragic Rectocolitis, Biological Biermer’s Disease. *Transfusion Clinique et Biologique*.

[19] Giuffrida G., Markovic U., Nicolosi D., Calafiore V. (2020). A Rare Case of Acquired Factor V Inhibitor, During Treatment With Dabigatran for Chronic Atrial Fibrillation, Successfully Treated With Bypassing Agents. *Clinical Case Reports*.

[20] Yokota Y., Inatomi O., Nakagawa M. (2021). Acquired Coagulation Factor V Inhibitor That Was Successfully Treated With Oral Corticosteroid Therapy. *Internal Medicine*.

[21] Kida W., Nakaya M., Ito A., Kozai Y., Bingo M. (2022). A Case of Acquired Factor V Inhibitor Following Nivolumab Administration. *Cureus*.

[22] Sei M., Mizuguchi M., Yagi H. (2022). Clinical Features of Five Cases of Acquired Factor V Deficiency. *Rinsho Ketsueki*.

[23] Katsuren E., Kohagura K., Kinjyo T. (2022). Acquired Factor V Inhibitor With Erythema and Eosinophilia in a Patient With End-Stage Renal Disease. *CEN Case Reports*.

[24] Arakaki S., Ono S., Kawamata F. (2023). Fatal Acquired Coagulation Factor V Deficiency after Hepatectomy for Advanced Hepatocellular Carcinoma as a Possible Immune Checkpoint Inhibitor-Related Adverse Event: A Case Report. *Surgical Case Reports*.

[25] Yu N., Liu X., Liu C., Tian W., Li W., Liu Y. (2023). Rare Acquired Factor V Inhibitors Combined With Positive Lupus Anticoagulant That Successfully Treated by Plasmapheresis and Prednisone Acetate: A Typical Case Report. *Annals of Hematology*.

[26] Ceglédi A., Dolgos J., Fekete M. (2023). Delayed Spontaneous Remission of Acquired Factor V Inhibitor Refractory to Immunosuppressive Therapy With Pregnancy-Associated Improvement. *Pathology and Oncology Research*.

[27] Ou K., Arakawa H., Togashi Y., Fujita H., Matsukura S. (2024). A Case of Acquired Factor V Inhibitor During Bullous Pemphigoid Treatment. *Cureus*.

[28] Ansari F., Lee Y., Ansari U., Kim P. (2024). Acquired Factor V Inhibitor Treated With Rituximab. *BMJ Case Reports*.

[29] Osaki T., Souri M., Ichinose A. (2022). Plasma Proteomics Associated With Autoimmune Coagulation Factor Deficiencies Reveals the Link Between Inflammation and Autoantibody Development. *International Journal of Hematology*.

[30] Taniwaki M., Katsutani S., Yamasaki M. (2019). Acquired Factor V Inhibitor After Antibiotic Treatment in a Patient With Pneumonia: A Case Report. *Annals of Hematology*.

[31] Wu M. T., Pei S. N. (2010). Development of Cephradine-Induced Acquired Factor V Inhibitors: A Case Report. *The Annals of Pharmacotherapy*.

[32] Mumford A. D., Ackroyd S., Alikhan R. (2014). Guideline for the Diagnosis and Management of the Rare Coagulation Disorders: A United Kingdom Haemophilia Centre Doctors’ Organization Guideline on Behalf of the British Committee for Standards in Haematology. *British Journal of Haematology*.

[33] Chandela M., Saxena A. K., Mehta R. K. (2024). Use of Tranexamic Acid in SARS-COV-2: Boon or Bane?. *Archives of Razi Institute*.

[34] Au W. Y., Lam C. C. K., Kwong Y. L. (2004). Successful Treatment of Acquired Factor VIII Inhibitor With Cyclosporin. *Haemophilia*.

[35] Wano Y., Kang Y., Masaki H. (2005). Cyclosporine A as an Effective Treatment for a Patient With Acquired Hemophilia A Complicated With Diabetes Mellitus and Ischemic Heart Disease. *Rinsho Ketsueki*.

